# Family Acculturation in Host and Immigrant Couples: Dyadic Research in an Italian Context

**DOI:** 10.5964/ejop.v14i4.1553

**Published:** 2018-11-30

**Authors:** Nadia Rania, Laura Migliorini, Stefania Rebora

**Affiliations:** a Department of Education Sciences, University of Genoa, Genoa, Italy; Department of Psychology, Webster University Geneva, Geneva, Switzerland; London School of Economics, London, United Kingdom

**Keywords:** family acculturation strategies, acculturation attitudes, couples, acculturation domains, dyadic analyses

## Abstract

The purpose of this research is to study acculturation strategies and attitudes in central and peripheral domains of host and immigrant couples in an Italian context. The participants were 60 dyads (30 host couples and 30 immigrant couples) who completed a questionnaire based on the Relative Acculturation Extended Model (RAEM). Based on the analysis, we found that the general acculturation attitude preferred by immigrant couples is integration, and Italian couples prefer that immigrants adopt it. Furthermore, Italian partners show moderate internal agreement, whereas immigrant couples show a high degree of agreement. In both groups, the level of agreement between dyadic members is only partially determined by their membership within a social group. The socio-cultural context has a significant role in the internal similarity of Italian couples. In contrast, there is dyadic agreement within immigrant couples.

Over the last thirty years Europe has been presented with continuous immigration, becoming an increasingly multicultural context in which people are in contact with other cultures daily (Cushner, 2008; Rania, Migliorini, Cardinali, & Rebora, 2015; Rania, Migliorini, Rebora, & Cardinali, 2014) and where many migrants experience increasing pressures to assimilate to their host culture. The acculturation process begins as soon as new immigrants enter the country and involves numerous changes in the use of language, behaviours, attitudes, and values. Research on acculturation has identified several acculturation models, most commonly used is Berry’s bidimensional model (Berry, 1980, 1997), which defines four acculturation outcomes: assimilation (high acquisition, low maintenance), separation (low acquisition, high maintenance), marginalization (low acquisition, low maintenance), and integration (high acquisition, high maintenance). However, recent models, that we will explain later, have underlined that the acculturation process is complex, relative and specific. Arends-Tóth and Van de Vijver (2003) affirmed that an individual’s preference for adjustment or maintenance could vary according to the life subdomain or situation. Based on this perspective, Navas Luque et al. (2005) proposed a model in which there are acculturation options in different areas of life (work, economic, social, family, religious beliefs and customs, and ways of thinking, principles and values) that can be preferred and adopted concurrently. Acculturation has been theorized more frequently as an individual process. However, there could be different acculturation positions within families that could be negotiated among members because acculturation is a fluid and mutual process (Buckingham & Brodsky, 2015). Dumka and Roosa (1997) have already suggested that acculturation processes change for each person or family, and they should not relate to the length of time in a host country or generational status. The family approach is relevant because acculturation can be considered a process that involves both individuals and groups. In fact, the family represents the interface between individuals and society. Immigrant families need both to maintain their own identities and culture and be open to the demands of the new context. The challenges faced by immigrant families are to find a balance between protective and emancipative functions. As a resource for wellbeing, the first function aims to maintain patterns of reference to ensure continuity with the history and memory of family values, while the second function is related to the exploration of the new environment, its social and cultural characteristics, and the onset of new relationships (Falicov, 2003). In the literature, family acculturation has been conceptualized as a long-term process, during which family members are integrated into the host society (Berry, 2005; Carranza & Turner, 2003). Researchers of family acculturation in particular have studied child-parent relationships (Dinh & Nguyen, 2006; Kim, Chen, Li, Huang, & Moon, 2009; Tardif-Williams & Fisher, 2009), adolescent-parent relationships (Martinez, 2006; Smokowski, Rose, & Bacallao, 2008), acculturation stress (Hurwich-Reiss & Gudiño, 2015), second generations (Sabatier & Berry, 2008), family outcomes of acculturation in relation to children (Phinney, Kim-Jo, Osorio, & Vilhjalmsdottir, 2005; Phinney, Ong, & Madden, 2000) and the acculturation gap. As underlined by Stuart, Ward, Jose, and Narayanan (2010), parents and adolescents differ in their expectations in relevant domains such as privacy, trust, and relationships. These differences that are present in the normal development processes of all families could lead to intergenerational conflict in family migration, intensified by acculturation processes. Specifically, most studies that focus on the intergenerational acculturation gap have underlined that parents and children adapt asynchronously to the new culture, thereby resulting in acculturation gaps between the two generations (Kuczynski, Navara, & Boiger, 2011). Moreover, children in particular may adjust to the new context more swiftly than parents (Buckingham & Brodsky, 2015), thus creating intergenerational conflicts, stress, and problems of adaptation to the behaviour of the various components of the family. Recently, some researchers have considered the acculturation process within a familial context; however, studies that have considered multiple members simultaneously are sporadic (Stuart, Ward, Jose, & Narayanan, 2010). Therefore, Dinh and Nguyen (2006) have suggested that research should focus on the acculturation experience of the family as a whole. In the field of acculturation psychology, there are numerous studies documenting the impact of changes in the cultural context on parental socialization practices and parent-child relationships (Bornstein & Cote, 2006a, 2006b; Carra, Lavelli, Keller, & Kärtner, 2013; Durgel, Leyendecker, Yagmurlu, & Harwood, 2009; Moscardino, Nwobu, & Axia, 2006; Raghavan, Harkness, & Super, 2010; Rania, Migliorini, & Rebora, 2016; Tajima & Harachi, 2010; Yagmurlu & Sanson, 2009). A number of scholars has pointed out that the attitudes, values and behaviours of immigrant families are affected by acculturation processes and integration with the receiving culture (Berry, 1997; Bornstein & Cote, 2006a; Durgel et al., 2009; Tajima & Harachi, 2010; Yagmurlu & Sanson, 2009). Even native families are involved in a process of mutual exchange that occurs when different cultural groups come into contact with one another (Van Oudenhoven & Ward, 2013). In fact, acculturation processes produce changes in both the immigrant and native populations (Larsen, Vazov, Krumov, & Schneider, 2013; Migliorini, Rania, & Cardinali, 2016). An analysis of previous literature shows that there are no studies on applications of acculturation models in the family context. In fact, the literature has mainly studied individual acculturation models applied to the parent-child relationship, while an analysis of the dyadic dimension applied to the parental couple represents an important novelty in the study of acculturation, which the present study intends to investigate. Therefore, in this article, we build on previous research in this area in four significant ways. First, we study the acculturation process at the family level, by focusing on couple dyads. Second, we adhere to the current views of the two-dimensional acculturation model, and then we apply this perspective on a dyadic level by considering the parental couple. Third, we use the dyadic level analysis of a parenting couple in the migration process and compare natives and immigrants. Finally, we emphasize that there are different ways of acculturation with respect to central life domains and peripheral life domains and therefore there could be different agreements within couples and consequently at the family level.

## Aims

The aims of the present research are to study the acculturation process of Italian and immigrant families. Specifically, this study analyzes general acculturation attitudes and strategies and attitudes in life domains using a family approach in host and immigrant couple dyads (married or cohabiting) with children in early childhood.

The aims of the present study are as follows:

1. to describe the general acculturation attitudes and life domain strategies and attitudes adopted by immigrant dyads and the perceptions of host dyads regarding the strategies and attitudes adopted by immigrant dyads;
2. to analyse the internal dyadic accord in Italian and immigrant couples with respect to acculturation strategies and attitudes;
3. to verify if the dyadic agreement between the two members of a couple is effective and not determined by membership of the social groups of women and men;
4. to study if the dyadic agreement diminishes once the stereotypical effect has been controlled for, because the socio-cultural context has a significant role in the internal similarity of the dyads.

## The Italian Context

Based on the migration flows towards Italy in recent years, the number of immigrants has greatly increased. The regular resident population in Italy in 2014, according to ISTAT, included almost 5 million foreigners. Over the past 10 years according to the results of the 15th general census of 2011 (ISTAT, 2013), the Italian population increased by 2.7 million people. This phenomenon appears to be attributable to the increase in foreign nationals to 5,029,000 as of January 1, 2017, equal to 9.05% of the total Italian population, which is estimated to be 60,579,000. The ISTAT (2017) report also shows that a change occurred in the last twenty years in migration flows in terms of the heterogeneity of backgrounds and the stabilization of immigrant presence in the territory. Indicators of this phenomenon are the increase in indefinite resident permits and acquisition of citizenship by naturalization, specifically, transmitted from parents to children and reaching adult age for those born in Italy. The statistics also report the strong tendency of immigrants to recompose their family of origin in Italy since they often have children who were previously born in the country of origin and decide to extend the family by having children in Italy. Furthermore, recent statistics on Italian family reunions state that most of the migratory movements are tied to reasons concerning the family.

## Method

### Participants

Participants included 60 dyads, 30 host couples and 30 immigrant couples living in two regions of central and northwestern Italy. Italian men had an average age of 39.2 years (range 27-60, *SD* = 11.98), while women had an average age of 39.9 years (range 29-65, *SD* = 6.58). Their children (46.7% male, 53.3% female) had an average age of 6.03 years. The most common levels of educational attainment among Italian women were post-university courses (23.3%) and high school diplomas (23.3%), while for men high school diplomas (33.3%) and university degrees (26.7%) were most common. In host couples women were mainly employed as clerical workers (33.3%), followed by teachers (16.7%) and housewives (16.7%). Among men, self-employment was most common (26.7%), followed by clerical workers (23.3%) and labourers (20%). As for immigrant couples, men had an average age of 33.43 years (range 27-60, *SD* = 13.53), while women had an average age of 31.53 years (range 24-44, *SD* = 10.17) and their children (60% males and 40% females) had an average age of 5.83 years. Immigrant couples had been in Italy for approximately 10 years. The most present cultural groups were Romanian (31.9%), Ecuadorian (28.6%), and Albanian (9.9%), and other groups were represented at a very low percentage, less than 4%. The most common level of educational attainment among immigrant couples was a high school diploma (women 35.7%, men 40%), followed by two or three years of high school (women 21.4%, men 30%), middle school completion (women 14.3%, men 10%) and university degrees for women (14.3%), whereas only 3.3% of men had obtained this degree. As for work, women were most commonly domestic helpers (27.6%) and 20% were housewives, followed by clerical workers (20%). Most men worked as labourers (63.3%).

### Measures

Among the different acculturation models present in the literature, it was decided to use the Relative Acculturation Extended Model (RAEM; Navas Luque, Garcia, & Rojas, 2006; Navas Luque, Rojas, Garcia, & Pumares, 2007; Navas Luque & Rojas, 2010), which addresses both acculturation strategies and acculturation attitudes. The acculturation strategies consider the real plan/situation for each domain, specifically the acculturation options that immigrants say they put into practice within the new society versus those that the natives perceive as being put into practice by immigrants. The acculturation attitudes refer to the ideal plan, that is, the acculturation options that immigrants would choose to put into practice if they could and that natives would prefer immigrants to use to integrate into the host community. The questionnaire was translated into Italian and then adjusted for an Italian context. In Table 1, the questions that were in the two versions of the questionnaire for Italians and immigrants are presented.

*General acculturation attitudes* were measured based on the of two questions described in the table below; the first related to the maintenance of the culture of the country of origin, and the second on the adoption of the host culture. Participants had to indicate the degree of their *agreement* (5) or *disagreement* (1) on a 5-point Likert scale.

*Acculturation strategies and attitudes in specific domains.* The questions related to this area were applied in each of the following life domains: work, economic, social, family, religious beliefs and customs, and ways of thinking, principles and values. The answer was given on a Likert scale from 1 = “*not at all*” to 5 = “*a lot*”.

The combination of the questions presented in Table 1 with these two-by-two questions led us to four different types of strategies (Navas Luque et al., 2005; Navas Luque, Garcia, & Rojas, 2006; Navas Luque, Rojas, Garcia, & Pumares, 2007; Navas Luque, Rojas, Pumares, Lozano, & Cuadrado, 2010).

**Table 1:** Questions in the Italian and Immigrant Versions of the Questionnaire

Italian version	Immigrant version
General acculturation attitudes	
"Italians should let immigrants live in this country according to their customs".	“People of your country of origin should seek to live in Italy according to Italian custom".
“Italian customs should let immigrants participate fully in the life of this society".	"People of your country of origin should seek to participate fully in the life of Italian society".
Acculturation strategies (real plan) by specific domains	
"To what extent do you believe that immigrants maintain the traditions from their original culture”.	“To what extent do you maintain your original culture”.
"To what extent do you believe that immigrants have adopted Italian culture”.	“To what extent have you adopted the host culture”.
Acculturation attitude (ideal plan) by specific domains	
“To what extent would you like immigrants to keep their original culture”.	“To what extent would you like to maintain your original culture”.
“To what extent would you like immigrants to adopt Italian culture”.	“To what extent would you like to adopt Italian culture”.

### Procedure

The participants were contacted through their children’s teachers, and a letter described the aims of the study. Then, they met the researchers and completed the self-report questionnaire. An assistant researcher was present while the questionnaire was being completed to supervise data collection. Instructions were given at the start of the study informing people that participation was voluntary, and that responses were confidential and anonymous in compliance with the Italian law on privacy (No.196/2003). The procedures fully complied with the research ethical code of the Italian Association of Psychology, and the informed consent protocol was provided to participants.

### Data Analysis

Data were analysed using SPSS software for statistical analysis, version 18. To describe the types of general acculturation attitudes, mean scores and standard deviations were calculated. To integrate individuals into one of four general strategies, in line with the work of Mancini and Bottura (2014) and Navas Luque et al. (2010), single sample t-tests were run for each answer using the average value of 3. Scores below 3 for both responses indicated the adoption of a strategy/attitude of marginalization; if the score for the first question was higher than 3 and the score for the second question was lower than 3, the strategy/attitude was separation; if the score for the first question was less than 3 and the score for the second question was higher than 3, the strategy/attitude was assimilation; if the score for both questions was higher than 3, a strategy of integration was indicated; if the score for one or both questions was equal to 3, the responses were categorized as indeterminate, as proposed by Navas Luque et al. (2010). To describe acculturation strategies and attitudes for domains in Italian and immigrant dyads, average scores and standard deviations were calculated. The data analysis of dyads includes several techniques that enable considering the complexity of family relationships and their interdependence. Lanz and Rosnati (2002) argue that in the literature, there is no score that represents the complexity of family relationships. For this reason, researchers usually use multiple dyadic indices to compare them (Fisher, Kokes, Ransom, Philips, & Rudd, 1985). The differences between host and immigrant dyads were calculated by independent sample t-tests. Furthermore, paired samples t-tests to analyse the internal differences of the members of the dyads of both groups were performed. The effect size was described with Cohen's *d* coefficient.

The dyadic correlations (*r_dyadic_*) and the index discrepancy (*d*) were also used. Dyadic correlations were used to detect the degree of similarity within the dyad (Lanz & Rosnati, 2002), considering the preservation of the culture of origin and the adoption or maintenance of the host culture in peripheral and central domains. The discrepancy index was calculated to detect the level of disagreement between the two members of the dyad on the same constructs. For dyadic correlations, a correlation index was applied, according to Alfieri and Marta (2012). The correlation index used in the present work was Pearson’s, which enables verifying if the constructs are specific to the social groups to which they belong (women and men) or to dyads. In fact, a significant correlation indicates that a particular construct is specific to the social groups and not to the dyad (women and men of the same pair); conversely, a low correlation indicates the specificity of that size to the dyad.

To measure the stereotypical effect, the following procedure proposed by Kenny and Acitelli (1994) was applied: the average of the responses of all husbands (MH1) on the same item or that of wives (MW1) on the same item was subtracted from the husband's response (H1) and the wife’s response (W1) to each individual item of a scale, respectively. Therefore, MH1 and MW1 represented the stereotypes of the response of the husbands and the wives, respectively. Before calculating the dyadic correlation, we subtracted MH1 or MW1 from the item. The differences between the scores of the Italian and immigrant dyads on the scale were calculated by independent samples t-test. The effect size was described with Cohen's d coefficient. Comparisons between the average dyadic scores (*r_dyadic_*) and dyadic cleaned scores (*r _"pure"_*) were calculated using paired samples t-tests.

## Results

**Figure 1:**
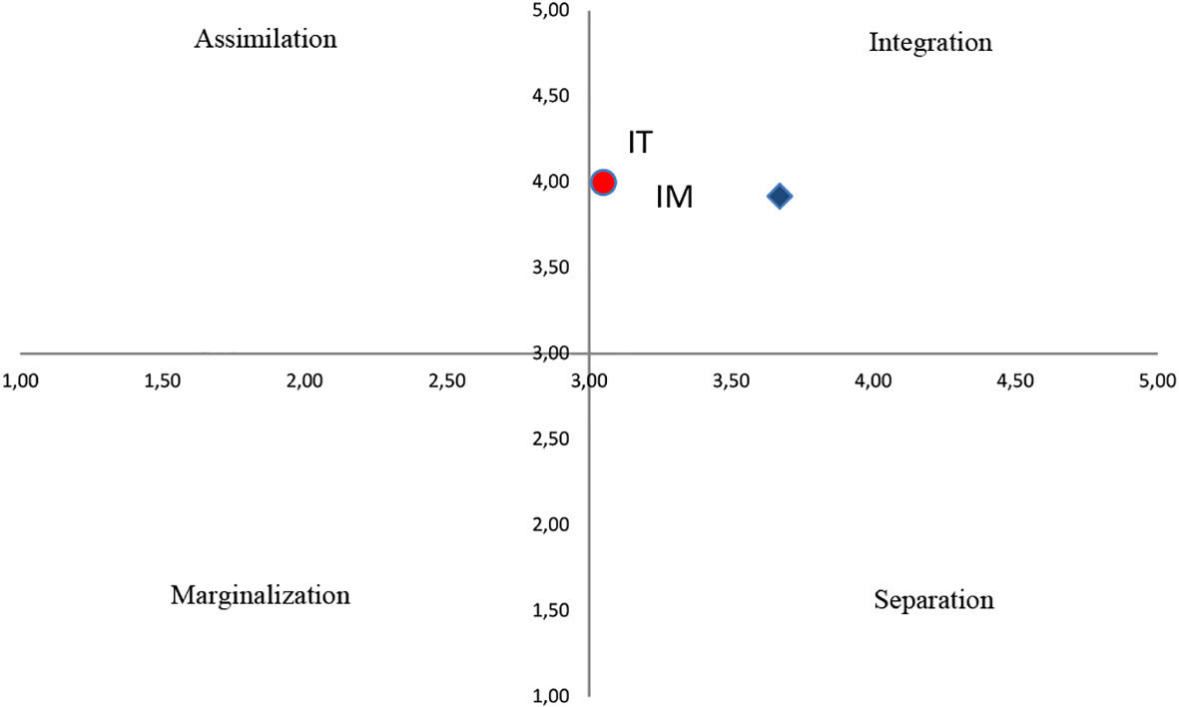
General acculturation attitudes in Italian and immigrant dyads. *Note.* IT = Italian dyads; IM = immigrants dyads.

Figure 1 shows the scores on general acculturation attitudes that, according to the Italian dyads, are adopted by immigrants and those that immigrant dyads claim to adopt. The figure was generated by combining the scores on the two main questions described above in the instrument section according to Berry’s model (1980). Specifically, it is a two-dimensional model that describes the four acculturation outcomes: assimilation, integration, marginalization and separation.

For both groups of participants, we reported the deviation from the mean value (3) of the average scores for the two questions about general acculturation attitude. The change in mean scores of Italians was significant only in relation to the adoption of Italian culture by immigrants (question 2 = 4.00, *t*(29) = 8.02, *p* < .001), indicating a preference towards integration. In regards to the immigrant dyads, the variation of the average scores of the two questions with respect to the mean value of the scale (3) was statistically significant (Question 1 = 3.67, *t*(29) = 3.88; Question 2 = 3.92, *t*(29) = 9.25), and the data indicated a preference for integration.

In line with the literature on acculturation models for domains (Arends-Tóth & Van de Vijver, 2003) applied to the RAEM model by Mancini and Bottura (2014) and Mancini, Navas Luque, López-Rodríguez, and Bottura (2018), the domain-specific strategies and the acculturation attitudes of dyads were presented by aggregating them into peripheral (work, economic, social) and central domains (family, religious beliefs and customs, and ways of thinking, principles and values). In Table 2, we report the mean scores of Italian and immigrant dyads and the significant differences between them. In regard to the real plan it seemed that the Italian dyads perceived the tendency of immigrants to maintain their culture of origin in the central domains of life (*M* = 8.4) and their tendency to adopt Italian culture in the peripheral domains (*M* = 3.10). These data were supported by immigrant dyads whose members confirmed that they maintain their own culture in the central domains (*M* = 4.22) of life and adopted Italian culture in the peripheral domains (*M* = 3.92).

**Table 2:** Descriptive Analysis of Italian and Immigrant Dyads Regarding Acculturation Strategies and Attitudes for Peripheral and Central Domains

Domains	Dyads	*n*	*M*	*SD*	*t*	*df*
*Real Plan - Acculturation strategies*						
Maintenance Peripheral					-0.84	58
	IT	30	3.39	0.62		
	IM	30	3.53	0.69		
Maintenance Central					-0.84	58
	IT	30	4.08	0.57		
	IM	30	4.22	0.65		
Adoption Peripheral					-5.65***	58
	IT	30	3.10	0.54		
	IM	30	3.92	0.57		
Adoption Central					-5.26***	58
	IT	30	2.27	0.60		
	IM	30	3.43	1.05		
*Ideal Plan - Acculturation attitudes*						
Maintenance Peripheral					-1.47	58
	IT	30	3.32	0.62		
	IM	30	3.58	0.72		
Maintenance Central					-2.45*	58
	IT	30	3.45	0.68		
	IM	30	3.97	0.95		
Adoption Peripheral					0.00	58
	IT	30	3.77	0.64		
	IM	30	3.77	0.81		
Adoption Central					-0.46	57
	IT	29	3.05	0.84		
	IM	29	3.17	0.82		

*Note*. Significant differences resulted from the *t*-test for independent samples between Italian and immigrant dyads.

*p < .05. ***p < .001.

In regards to the ideal plan, Italian dyads preferred that immigrants adopted the Italian culture in the peripheral domains of life (*M* = 3.77) and maintained their own culture in the central domains (*M* = 3.45), as confirmed by the immigrant dyads that reported a higher preference for maintaining their culture of origin in the central domains of life (*M* = 3.97) and for adopting Italian culture in the peripheral domains (*M* = 3.77). Significant differences emerged regarding the real plan in the adoption of the culture of the host country in the peripheral domains of life (*t*(58) = -5.65, *p* < .001. Cohen's *d* = 1.48), in the central domains (strategies) (*t*(58) = -5.26, *p* < .001, Cohen's *d* = 1.36), and in the central domains of the ideal plan concerning the maintenance of their culture of origin (attitudes) (*t*(58) = -2.45, *p* < .05, Cohen's *d* = 0.63).

To verify the level of agreement between the partners of the dyads in relation to the investigated variables, we calculated dyadic correlations (Lanz & Rosnati, 2002). Table 3 shows the scores of internal agreement with the dyads (*r*_dyadic_), the discrepancy index (d) and the Pearson correlation.

**Table 3:** Dyadic Correlations, Pearson Correlations and Discrepancy Index for Italian and Immigrant Dyads

Domains	Dyads IT			Dyads IM		
	*r*_dyadic_	*d*	*r*	*r*_dyadic_	*d*	*r*
Real Plan - Acculturation strategies						
Maintenance Peripheral	.81	.62	.48	.74	.56	.51
Maintenance Central	.16	.59	.18	.67	.43	.84
Adoption Peripheral	.45	.68	.56	.79	.46	.38
Adoption Central	.47	.73	.36	.45	.54	.53
Ideal Plan - Acculturation attitudes						
Maintenance Peripheral	.62	.84	.43	.77	.52	.58
Maintenance Central	.36	1.00	.14	.57	.63	.45
Adoption Peripheral	.60	.77	.33	.72	.55	.54
Adoption Central	.46	.93	.36	.69	.56	.61

Regarding the real plan for the Italian dyads, the level of agreement between the members was moderate, with the exception of the perception of maintenance of culture of origin by immigrants in peripheral domains, which had a high amount of dyadic agreement, and with the exception of the perception of maintaining the culture of origin by immigrants in central domains, for which the level of dyadic agreement was very low. Therefore, the discrepancy index was reported to be moderate although it trended towards 1, which indicated a lack of internal agreement in dyads.

The difference between the dyadic relationship and the index of discrepancy scores was attributable to differences between the mean scores and the standard deviations between the members of dyads. The Pearson correlations of the perception of Italians maintenance of the culture of origin by immigrants were moderate and significant compared to the perception of what immigrants should adopt of the culture of the receiving country. In the peripheral domains of life, the correlation was high and significant, indicating a specificity for social groups (women and men) and not for dyads, whereas in the central domains it was moderate and not significant, indicating a specificity for the dyads.

Regarding the ideal plan, the dyadic correlation showed that there was a low level of agreement between partners in relation to the perception that immigrants desire to retain their culture of origin and to adopt the culture of the dominant society in the central domain, whereas there was good agreement regarding the perception that immigrants desire to maintain their culture and to adopt Italian culture in the peripheral domains. The values of the discrepancy index were high. In sum, the members of Italian dyads showed a level of agreement that ranged from moderate to low. Regarding the ideal plan of the desire of immigrants to maintain their culture in peripheral domains of life, the correlation between Italian women and men was strong and significant, indicating the specificity of the social groups (women and men) and not the dyads; however, the desire of immigrants to adopt Italian culture in the central domains of life and adopt Italian culture in the peripheral domains had a moderate and not significant relation. A weak and not significant relationship emerged concerning the desire to maintain the culture of origin in the central domains, which indicated a dyadic specificity with respect to this dimension. In general, the agreement between members was greater and more specific to dyads in the central domains of life than in the peripheral domains.

Immigrant dyads showed a good degree of agreement in the real and ideal plans; in fact, the correlations of the values of the dyads were high on all dimensions. Moreover, to confirm this, immigrant dyads had a discrepancy index at a medium level for the real plan, whereas for the ideal plan, the discrepancy index was closer to 1, indicating a trend towards agreement within dyads. Therefore, in general, partners within immigrant dyads seem to have a high level of agreement. As for the Pearson correlations of immigrant dyads, the real plan dimensions were all characterized by strong and significant relations, and with respect to the adoption of Italian culture in the peripheral domains of life, a moderate and significant relationship was found. Regarding the ideal plan, the trend of the correlation was the same; in fact, all dimensions had strong and significant correlations, with the exception of the preference to maintain Italian culture in the central domains of life, for which the relationship was moderate and significant. Consequently, from these results it seems that agreement between immigrant women and men was primarily related to a social factor (belonging to groups of women and men). Only the maintenance of Italian culture in the central domains in the real plan was specific to the dyad. For the real plan, internal agreement between the members was high, and it was in the moderate range only in the case of the adoption of Italian culture in the central domains of life. However, for the ideal plan, the agreement was high in all dimensions, showing a high level of similarity between immigrant partners. To confirm that the level of agreement among members of a dyad was not largely due to the cultural context in which they live, we performed a comparison between the dyadic score and the dyadic score *r*_“pure”_, the score from which the stereotypical effect was subtracted (Table 4).

**Table 4:** Comparisons Between Average Scores of Total Dyadic Correlations and Pure Scores for Italian and Immigrant Dyads

Domains	Dyads IT				Dyads IM			
	*r*_dyadic_	*r*_“pure”_	*t*	*df*	*r*_dyadic_	*r*_“pure”_	*t*	*df*
Real plan - Acculturation strategies								
Maintenance Peripheral	.81	.42	3.47**	49	.74	.53	1.81	23
Maintenance Central	.16	.32	-0.78	3	.67	.74	-0.80	15
Adoption Peripheral	.45	.39	0.55	18	.79	.71	1.43	17
Adoption Central	.47	.20	1.45	8	.45	.63	-1.35	18
Ideal plan - Acculturation attitudes								
Maintenance Peripheral	.62	.39	1.91	19	.77	.58	1.56	18
Maintenance Central	.36	.51	-0.86	7	.57	.50	0.47	20
Adoption Peripheral	.60	.34	1.53	19	.72	.55	1.32	20
Adoption Central	.46	.54	-0.44	11	.69	.55	0.84	13

**p < .01.

The levels of internal similarity in the host dyads for the real plan were high compared to the perception of immigrants’ maintenance of their culture in peripheral domains. With regard to the adoption of Italian culture by immigrants both in the peripheral and central domains, the levels of internal similarity were moderate; the relationship decreased considerably with respect to the perception of immigrants’ maintenance of their culture in the central domains of life. For the ideal plan, the level of internal agreement of Italian dyads was high concerning the peripheral domains of life and whether they preferred immigrants to retain their culture or adopt the Italian culture; however, there was a moderate level of internal agreement regarding to the central domains of life both in the preference that immigrants adopt Italian culture and in the preference to maintain their own culture.

After calculating the stereotypical effect, the scores decreased for the real plan relative to the maintenance of their culture in peripheral domains of life, and in the adoption of Italian culture both in the peripheral and central domains. These results are characteristic of the social groups (host and immigrants) to which the dyads belong.

On the contrary, the score for maintaining the culture of origin in the central domains of life increased and was specific to the agreement between dyad partners; the scores decreased for the ideal plan with regard to the desire that immigrants adopt Italian culture and maintain their culture of origin in peripheral domains of life, thus being specific to the social group to which they belonged (host and immigrants). Conversely, the scores increased when compared to the desire that immigrants maintain their culture or adopt the Italian culture in the central domains. These results would be specific to the level of agreement between dyad partners.

By carrying out the paired samples t-test between the dyadic correlations and the correlations and correcting for the stereotypical effects, significant differences emerged for the real plan regarding the dimension of the perception of immigrants’ maintenance of their own culture in peripheral domains of life (*t*(25) = 3.47, *p* < .01). Shifting the focus to the immigrant dyads, in regards to the real plan, the levels of similarity between the members of a dyad were high, except for the adoption of Italian culture in the central domains of life which was moderate. Regarding the ideal plan, agreement was high in all dimensions. After calculating the stereotypical effect for the real plan, the scores related to the maintenance of their culture and the adoption of Italian culture in the peripheral domains were lower. On the contrary, the scores increased relative to the maintenance of their culture and the adoption of Italian culture in the central domains, highlighting the specificity of these dimensions to the level of agreement between dyad partners. Instead, regarding the ideal plan, the scores were low for all dimensions. Although the scores for immigrant dyads decreased, they were still higher than .50 after the removal of the stereotypical effect, indicating high internal agreement for dyads for all the RAEM’s dimensions. Since there was significant agreement between the members, there were no significant differences when comparing the averages for paired samples between the dyadic correlation and the correlation corrected for the stereotypical effect.

## Discussion and Conclusions

This study aimed to describe the general acculturation attitudes and acculturation strategies and attitudes in the peripheral and central domains of life from a family perspective in host and immigrant dyads. In line with previous studies conducted at the individual level (Berry, 1997; Navas Luque et al., 2006; Van Oudenhoven et al., 1998; Zagefka & Brown, 2002), the general acculturation attitude preferred by immigrant dyads was integration. The Italian dyads, in line with the immigrant dyads, perceived that immigrants preferred the option of integration, although there was an evident tendency towards assimilation. Regarding the perceptions of the host culture, Zagefka, Tip, Gonzales, Brown, and Cinnirella (2012) explain how it is possible that local residents, perceiving the intention of immigrants to adopt their culture, are more willing to open up to them.

The present research offers a relevant contribution to the acculturation literature by exploring the acculturation practices of families in an intercultural context. The analysis of the dyadic dimension represents an important novelty in the studies on acculturation considering the close relationship between acculturation and family relationships, as the family influences the effects of migration on future generations. Namely, within the acculturation process, it is necessary to consider the contact within different family models that can influence one another in some dimensions of family life (Rania, Migliorini, Rebora, & Cardinali, 2015).

The present study also aimed to verify the existence of a dyadic agreement between the members of host couples and those of immigrant couples with respect to acculturation strategies and attitudes. Generally, the Italian dyads showed moderate internal agreement, whereas for immigrant dyads, a high degree of agreement between members was found. In fact, dyadic agreement could be considered support for the negotiation of acculturation strategies not only at the couple level but also at the family level (Kisselev, Brown, & Brown, 2010). The high degree of agreement in immigrant dyads could also be considered in line with the idea that the recognition and protection of family ties represent a focal point for immigrants (Regalia & Giuliani, 2014). As suggested by McGoldrick and Ashton (2012), migration is a factor that influences how family members relate to their cultural heritage, to others of their cultural group, and to the preservation of cultural traditions. However, a recent study by Migliorini, Rania, Tassara, and Cardinali (2016) points out that migrant families obtain lower scores than host dyads regarding ritual symbolic significance; this finding indicates that further studies are needed on this topic.

Another aim was to investigate whether the degree of dyadic agreement between the members of a couple was effective and not determined by membership of the social groups of men and women with respect to acculturation domains. In the present study, the Italian dyads showed a dyadic specificity regarding the central domains in life based on the real plan, and specificity for groups (women and men) regarding the peripheral domains. In relation to the ideal plan, dyadic specificity applied to the preference for adopting Italian culture both in the peripheral and central domains, but, for the central domains, dyadic specificity applied only to the preference that immigrants maintain their culture of origin. On the contrary, agreement regarding the preference of maintenance by immigrants of their culture of origin in the peripheral domains was specific to the groups.

Moreover, the immigrant dyads showed specific agreement between dyadic members only in the adoption of Italian culture in the peripheral domains of life, and agreement on all remaining dimensions seemed to be specific to the gender groups to which they belonged (men and women). According to Regalia and Giuliani (2014) these gendered differences are not uncommon and could be related to on various areas of life (values, practices, behaviours).

The final aim was to investigate whether the dyadic agreement would be decreased by controlling the stereotypical effect, specifically, whether the socio-cultural context played a significant role in the internal similarity of dyads, according to the methodology of dyadic analyses followed. Regarding the Italian dyads, after controlling for stereotypical effects on the dyadic agreement, what emerged was that the environment prevailed in defining the internal similarity between members. Immigrant dyads, however, did not seem to be influenced by the context, but the dyadic agreement was similar to the responses provided by dyad members. This finding could be explained from an intergroup perspective, underlining that coherence between individuals of a minority group would be essential to not lose their cultural specificity (Verkuyten & Martinovic, 2014).

An aspect that is not very thoroughly studied in the literature like the theme of family acculturation is a strong point of this study, it may prove to be a limitation because the lack of literature on the subject hinders an effective comparison with data from other similar research in terms of content and methodology. Furthermore, another possible limitation comes from the lack of consideration of the individual variables in determining host and immigrant acculturation preferences; these individual variables are presented in the literature, and include psychological issues, group and national identification (Mashuri, Burhan, & van Leeuwen, 2013; Nesdale & Mark, 2000; Verkuyten, & Martinovic, 2012) and, ethnic identity (Phinney, Horenczyk, Liebkind, & Vedder, 2001). Another limitation to this research is that, although the use of convenience sampling was adequate considering the explorative nature of this study (De Carlo & Robusto, 1996), it did not allow us to obtain a high degree of representativeness in the sample. For this reason, the results should be interpreted with caution as they cannot be generalized to the full range of host and migrant couples.

According to Kisselev, Brown, and Brown (2010), qualitative research is often culturally congruent and consequently could be an effectively used multimethod approach that allows us to understand and draw conclusions about immigrant families. Future research could more deeply investigate this issue by expanding the sample size and by considering other measures of individual, couple and family well being that could in some way affect the participants' dyadic agreement. Therefore, future studies should focus on the direct measurement of acculturation strategies and attitudes implemented and desired by the native population in the peripheral and central domains of life since in the present study, only their perception of acculturation processes affecting immigrants was assessed. Furthermore, researchers who are interested in the acculturation processes of parenting dyads should also consider sample those who have divorced or no longer share a partnership. These further investigations could lead to understanding if acculturation and couple agreement could be maintained when the relationship is broken. Moreover, it would be interesting for future research to consider immigrants who have lived in the host country different lengths of time, to understand the differences related to the length of residency. The immigrant participants in this study had lived in Italy for approximately ten years, which could be considered a long time during which they could have modified their level of dyadic acculturation agreement. This research would enable adding another piece to the complex picture of Italian intercultural relationships.

With the increase in migratory flows and family reunification in various European countries, the scientific community should continue to study the processes of acculturation at the parental couple level to understand developments at the family level and understand how these dyadic agreements have repercussions at the generational level (Fine & Fincham, 2013). Until now, studies have focused on the dyadic level between parents and children. It is important to underline that successful integration could be considered a function of both acculturation strategies at the individual, dyadic and group levels in different domains, and the policies and practices of the receiving culture (Sabatier & Berry, 2008) in terms of multiculturalism and/or intergroup relationships.

Practical implications should take into account the different couples and family models that come into contact with the migration processes (Singh & Dutta, 2010). In fact, we believe our research has important strengths and interesting findings that could have practical implications for family-based interventions in community enrichment and school programs to support continuity in migrant family identity (Cigoli & Scabini, 2006) and to promote the well-being of both native and immigrant family members.

## References

[r1] Alfieri S. Marta E. (2012). Family and social generation compared in terms of ethnic prejudice in young adults: A study with family triads. TPM. Testing, Psychometrics, Methodology in Applied Psychology, 19(1), 35–47. doi:. 10.4473/TPM19.1.3

[r2] Arends-Tóth J. Van de Vijver F. J. R. (2003). Multiculturalism and acculturation: Views of Dutch and Turkish-Dutch. European Journal of Social Psychology, 33, 249–266. doi:. 10.1002/ejsp.143

[r3] Berry, J. W. (1980). Acculturation as varieties of adaptation. In A. S. Padilla (Ed.), *Acculturation: Theory, models and some new findings* (pp. 9-25). Boulder, CO, USA: Westview.

[r4] Berry J. W. (1997). Immigration, acculturation and adaptation. Applied Psychology, 46, 5–43. doi:. 10.1111/j.1464-0597.1997.tb01087.x

[r5] Berry J. W. (2005). Acculturation: Living successfully in two cultures. International Journal of Intercultural Relations, 29, 697–712. doi:. 10.1016/j.ijintrel.2005.07.013

[r6] Bornstein, M. H., & Cote, L. R. (2006a). *Acculturation and parent–child relationships: Measurement and development.* Mahwah, NJ, USA: Lawrence Erlbaum Associates.

[r7] Bornstein, M. H., & Cote, L. R. (2006b). Parenting cognitions and practices in the acculturative process. In M. H. Bornstein & L. R. Cote (Eds.), *Acculturation and parent–child relationships: Measurement and development* (pp. 173–196). Mahwah, NJ, USA: Lawrence Erlbaum Associates.

[r8] Buckingham S. L. Brodsky A. E. (2015). “Our differences don’t separate us”: Immigrant families navigate intrafamilial acculturation gaps through diverse resilience processes. Journal of Latina/o Psychology, 3(3), 143–159. doi:. 10.1037/lat0000042

[r9] Carra C. Lavelli M. Keller H. Kärtner J. (2013). Parenting infants: Socialization goals and behaviors of Italian mothers and immigrant mothers from West Africa. Journal of Cross-Cultural Psychology, 44(8), 1304–1320. doi:. 10.1177/0022022113486004

[r10] Carranza, M., & Turner, J. (2003). *Family acculturation: Salvadorian mothers and their adolescent daughters in the Canadian context*. Paper presented at the Conference on International Migration in the Americas - Emerging Issues, York University, Toronto, Canada.

[r11] Cigoli, V., & Scabini, E. (2006). *Family identity: Ties, symbols and transitions.* Mahwah, NJ, USA: Lawrence Erlbaum Associates.

[r12] Cushner K. (2008). International socialization of young people: Obstacles and opportunities. International Journal of Intercultural Relations, 32(2), 164–173. doi:. 10.1016/j.ijintrel.2007.09.004

[r13] De Carlo, N. A., & Robusto, E., (1996). *Teoria e tecniche di campionamento nelle scienze sociali*. Milano, Italy: Led edizioni universitarie.

[r14] Dinh K. T. Nguyen H. (2006). The effects of acculturative variables on Asian American parent–child relationships. Journal of Social and Personal Relationships, 23(3), 407–426. doi:. 10.1177/0265407506064207

[r15] Dumka L. E. Roosa M. W. (1997). Risk, conflict, mother’s parenting and adjustment in low income, Mexican immigrant and Mexican American families. Journal of Marriage and the Family, 59, 309–323. doi:. 10.2307/353472

[r16] Durgel E. S. Leyendecker B. Yagmurlu B. Harwood R. (2009). Sociocultural influences on German and Turkish immigrant mothers’ long-term socialization goals. Journal of Cross-Cultural Psychology, 40, 834–852. doi:. 10.1177/0022022109339210

[r17] Falicov, C. J. (2003). Immigrant family processes. In F. Walsh (Ed.), *Normal family processes* (pp. 280-300). New York, NY, USA: Guilford Press.

[r18] Fine, M. A., & Fincham, F. D. (2013). *Handbook of family theories: A content-based approach*. New York, NY, USA: Routledge.

[r19] Fisher L. Kokes R. F. Ransom D. C. Philips S. L. Rudd P. (1985). Alternative strategies for creating relational family data. Family Process, 24, 213–224. doi:. 10.1111/j.1545-5300.1985.00213.x 4018242

[r20] Hurwich-Reiss E. Gudiño O. G. (2015). Acculturation stress and conduct problems among Latino adolescents: The impact of family factors. Journal of Latina/o Psychology. Advance online publication 10.1037/lat0000052

[r21] ISTAT. (2013). *Gli stranieri al 15° censimento della popolazione.* Retrieved from http://daticensimentopopolazione.istat.it

[r22] ISTAT. (2017). *La popolazione in Italia, nuove stime per l’anno 2016.* Retrieved from https://www.istat.it/it/files/2017/03/Indicatori-Demografici.pdf

[r23] Kenny D. A. Acitelli L. K. (1994). Measuring similarity in couples. Journal of Family Psychology, 8, 417–431. doi:. 10.1037/0893-3200.8.4.417

[r24] Kim S. Y. Chen Q. Li J. Huang X. Moon U. J. (2009). Parent–child acculturation, parenting, and adolescent depressive symptoms in Chinese immigrant families. Journal of Family Psychology, 23(3), 426–437. doi:. 10.1037/a0016019 19586205 PMC2746862

[r25] Kisselev P. Brown M. A. Brown J. D. (2010). Gender differences in language acculturation predict marital satisfaction: A dyadic analysis of Russian-speaking immigrant couples in the United States. Journal of Comparative Family Studies, 41(5), 767–782. Retrieved from http://www.jstor.org/stable/41604403.

[r26] Kuczynski, L., Navara, G., & Boiger, M. (2011). The social relational perspective on family acculturation. In S. S. Chuang & R. P. Moreno (Eds.), *Immigrant children: Change, adaptation, and cultural transformation* (pp. 171–192). Lanham, MD, USA: Lexington Books.

[r27] Lanz, M., & Rosnati, R. (2002). *Metodologia della ricerca sulla famiglia*. Milano, Italy: Led.

[r28] Larsen, K. S., Vazov, G., Krumov, K., & Schneider, J. F. (Eds.). (2013). *Advances in international psychology: Research approaches and personal dispositions, socialization processes and organizational behavior*. Kassel, Germany: Kassel University Press.

[r29] Mancini T. Bottura B. (2014). Acculturation processes and intercultural relations in peripheral and central domains among native Italian and migrant adolescents: An application of the Relative Acculturation Extended Model (RAEM). International Journal of Intercultural Relations, 40, 49–63. doi:. 10.1016/j.ijintrel.2013.12.002

[r30] Mancini T. Navas Luque M. López-Rodríguez L. Bottura B. (2018). Variants of biculturalism in migrant and host adolescents living in Italy and Spain: Testing the importance of life domains through the Relative Acculturation Extended Model. International Journal of Psychology, 53(S1), 71-80. doi:. 10.1002/ijop.12432 28488277

[r31] Martinez C. R. Jr (2006). Effects of differential family acculturation on Latino adolescent substance use. Family Relations, 55, 306–317. doi:. 10.1111/j.1741-3729.2006.00404.x

[r32] Mashuri A. Burhan O. K. van Leeuwen E. (2013). The impact of multiculturalism on migrant helping. Asian Journal of Social Psychology, 16, 207–212. doi:. 10.1111/ajsp.12009

[r33] McGoldrick, M., & Ashton, D. (2012), Culture: A challenge to concepts of normality. In F. Walsh (Ed.), *Normal family processes: Growing diversity and complexity* (4th ed., pp. 249-272). New York, NY, USA: Guilford.

[r34] Migliorini L. Rania N. Cardinali P. (2016). Acculturation strategies and adjustment in immigrant and host Italian communities. TPM. Testing, Psychometrics, Methodology in Applied Psychology, 23(1), 99–112. doi:. 10.4473/TPM23.1.7

[r35] Migliorini L. Rania N. Tassara T. Cardinali P. (2016). Family routine behaviours and meaningful rituals: A comparison between Italian and migrant couples. Social Behavior and Personality, 44(1), 9–18. doi:. 10.2224/sbp.2016.44.1.9

[r36] Moscardino U. Nwobu O. Axia G. (2006). Cultural beliefs and practices related to infant health and development among Nigerian immigrant mothers in Italy. Journal of Reproductive and Infant Psychology, 24(3), 241–255. doi:. 10.1080/02646830600821280

[r37] Navas Luque M. Garcia M. Rojas A. J. (2006). Acculturation strategies and attitudes of African immigrants in the south of Spain: Between reality and hope. Cross-Cultural Research, 40(4), 331–351. doi:. 10.1177/1069397105283405

[r38] Navas Luque M. Garcia M. C. Sanchez J. Rojas A. J. Pumares P. Fernandez J. S. (2005). Relative Acculturation Extended Model (RAEM): New contributions with regard to the study of acculturation. International Journal of Intercultural Relations, 29, 21–37. 10.1016/j.ijintrel.2005.04.001

[r39] Navas Luque, M., & Rojas, A. J. (2010). *Aplicacion del modelo ampliado de acculturacion relativa (MAAR) a nuevos collectivos de inmigrantes en Andalucia: Rumanos y ecuatorianos*. Sevilla, Spain: Direction general de coordinacion, Consejería de empleo, Junta de Andalucía.

[r40] Navas Luque M. Rojas A. J. Garcia M. Pumares P. (2007). Acculturation strategies and attitudes to the Relative Acculturation Extended Model (RAEM): The perspectives of natives versus immigrants. International Journal of Intercultural Relations, 31, 67–86. doi:. 10.1016/j.ijintrel.2006.08.002

[r41] Navas Luque M. Rojas A. J. Pumares P. Lozano O. M. Cuadrado I. (2010). Perfiles de aculturación según el Modelo Ampliado de Aculturación Relativa: Autóctonos, inmigrantes rumanos y ecuatorianos. Revista de Psicología Social, 25(3), 295–312. doi:. 10.1174/021347410792675624

[r42] Nesdale D. Mark A. S. (2000). Immigrant acculturation attitudes and host country identification. Journal of Community & Applied Social Psychology, 10, 483–495. 10.1002/1099-1298(200011/12)10:6<483::AID-CASP580>3.0.CO;2-0

[r43] Phinney J. S. Horenczyk G. Liebkind K. Vedder P. (2001). Ethnic identity, immigration, and well-being. The Journal of Social Issues, 57(3), 493–510. 10.1111/0022-4537.00225

[r44] Phinney J. S. Kim-Jo T. Osorio S. Vilhjalmsdottir P. (2005). Autonomy and relatedness in adolescent-parent disagreements: Ethnic and developmental factors. Journal of Adolescent Research, 20, 8–39. doi:. 10.1177/0743558404271237

[r45] Phinney J. S. Ong A. Madden T. (2000). Cultural values and intergenerational value discrepancies in immigrant and non-immigrant families. Child Development, 71(2), 528–539. doi:. 10.1111/1467-8624.00162 10834482

[r46] Raghavan C. S. Harkness S. Super C. M. (2010). Parental ethnotheories in the context of immigration: Asian Indian immigrant and Euro-American mothers and daughters in an American Town. Journal of Cross-Cultural Psychology, 41, 617–632. doi:. 10.1177/0022022110362629

[r47] Rania N. Migliorini L. Cardinali P. Rebora S. (2015). Giving a face to immigration and integration processes: The use of Photovoice with Italian young adults. Qualitative Report, 20(6), 780–798. Retrieved from http://www.nova.edu/ssss/QR/QR20/6/rania4.pdf

[r48] Rania N. Migliorini L. Rebora S. (2016). Parental competence in Italy: A comparison between Italian and immigrant parents. Marriage & Family Review, 54, 1-14. doi:. 10.1080/01494929.2016.1247760

[r49] Rania N. Migliorini L. Rebora S. Cardinali P. (2014). Enhancing critical dialogue about intercultural integration: The Photovoice technique. International Journal of Intercultural Relations, 41, 17–31. doi:. 10.1016/j.ijintrel.2014.06.006

[r50] Rania N. Migliorini L. Rebora S. Cardinali P. (2015). Daily family routines of Italian and Ecuadorian immigrant mothers in everyday life: A qualitative approach using diaries and interviews. SAGE Open, 5(4), 1–13. doi:. 10.1177/2158244015609411

[r51] Regalia, C., & Giuliani, C. (2014). Così lontani così vicini. La prospettiva psico-sociale nello studio delle famiglie migranti. In CISF (Ed.), *Le famiglie di fronte alle sfide dell’immigrazione* (pp. 153-179) Trento, Italy: Erickson.

[r52] Sabatier C. Berry J. W. (2008). The role of family acculturation, parental style, and perceived discrimination in the adaptation of second-generation immigrant youth in France and Canada. European Journal of Developmental Psychology, 5(2), 159–185. doi:. 10.1080/17405620701608739

[r53] Singh, R., & Dutta, S. (2010). *“**Race” and culture: Tools, technique and trainings: A manual for professionals.* London, United Kingdom: Karnac.

[r54] Smokowski P. R. Rose R. Bacallao M. L. (2008). Acculturation and Latino family processes: How cultural involvement, biculturalism, and acculturation gaps influence family dynamics. Family Relations, 57(3), 295–308. doi:. 10.1111/j.1741-3729.2008.00501.x

[r55] Stuart J. Ward C. Jose P. E. Narayanan P. (2010). Working with and for communities: A collaborative study of harmony and conflict in well-functioning, acculturating families. International Journal of Intercultural Relations, 34, 114–126. doi:. 10.1016/j.ijintrel.2009.11.004

[r56] Tajima E. A. Harachi T. W. (2010). Parenting beliefs and physical discipline practices among Southeast Asian immigrants: Parenting in the context of cultural adaptation to the United States. Journal of Cross-Cultural Psychology, 41(2), 212–235. doi:. 10.1177/0022022109354469

[r57] Tardif-Williams C. Y. Fisher L. (2009). Clarifying the link between acculturation experiences and parent–child relationships among families in cultural transition: The promise of contemporary critiques of acculturation psychology. International Journal of Intercultural Relations, 33, 150–161. doi:. 10.1016/j.ijintrel.2009.01.001

[r58] Van Oudenhoven J. P. Ward C. (2013). Fading majority cultures: The Implications of transnationalism and demographic changes for immigrant acculturation. Journal of Community & Applied Social Psychology, 23(2), 81–97. doi:. 10.1002/casp.2132

[r59] Van Oudenhoven J. P. Prins K. S. Buunk B. P. (1998). Attitudes of minority and majority members towards adaptation of immigrants. European Journal of Social Psychology, 28(6), 995–1013. doi:. 10.1002/(SICI)1099-0992(1998110)28:6<995::AID-EJSP908>3.0.CO;2-8

[r60] Verkuyten M. Martinovic B. (2012). Immigrant’s national identification, meaning, determinants, and consequences. Social Issues and Policy Review, 6(1), 82–112. 10.1111/j.1751-2409.2011.01036.x

[r61] Verkuyten, M., & Martinovic, B. (2014). Minority identity and host national identification among immigrants. In C. K. De Dreu (Ed.), *Social conflict within and between groups* (pp. 55-74). Hove, United Kingdom: Psychology Press.

[r62] Yagmurlu B. Sanson A. (2009). The role of child temperament, parenting and culture in the development of prosocial behaviors. Australian Journal of Psychology, 61, 77–88. 10.1080/00049530802001338

[r63] Zagefka H. Brown R. (2002). The relationship between acculturation strategies, relative fit and intergroup relations: Immigrant-majority relations in Germany. European Journal of Social Psychology, 32, 171–188. doi:. 10.1002/ejsp.73

[r64] Zagefka H. Tip L. K. Gonzales R. Brown R. Cinnirella M. (2012). Predictors of majority members’ acculturation preferences: Experimental evidence. Journal of Experimental Social Psychology, 48, 654–659. doi:. 10.1016/j.jesp.2011.12.006

